# Acoustic Emission Characteristics During Shear Failure of Active Waveguide Structure for Rock Slope Monitoring

**DOI:** 10.3390/s26144426

**Published:** 2026-07-12

**Authors:** Zhihui Wu, Lingjun Zhang, Jianjun Yang, Jie Dong, Yongxin Yu, Yunlong Sun

**Affiliations:** 1 College of Civil Engineering, Hebei University of Architecture, No. 13 Chaoyang Road, Zhangjiakou 075000, China; 15932112962@163.com (L.Z.); dongjie1003@hotmail.com (J.D.); 13049538240@163.com (Y.Y.); sunyunlong1105@163.com (Y.S.); 2 Hebei Provincial Key Laboratory of Civil Engineering Diagnosis, Renovation and Disaster Resistance, No. 13 Chaoyang Road, Zhangjiakou 075000, China; 3 Road & Bridge International Communication Teachnology Co., Ltd., No. 9, Lifu Street, Beixiaoying Town, Shunyi District, Beijing 101399, China; yangyuzhous@163.com

**Keywords:** rock slope monitoring, acoustic emission, active waveguide, evolutionary characteristics, experimental test

## Abstract

This study investigates the acoustic emission (AE) characteristics associated with the shear failure mode based on the principles of active waveguide monitoring for the bedding rock slopes. Physical simulation experiments were conducted to assess the AE response during the shear-induced failure process of active waveguide structures. The findings indicate that during the initial loading phase, the scatter points of the signals are concentrated within a relatively narrow range. As the shear stress exceeds 90% of the peak stress and approaches the failure stage, there is a significant increase in the AE count and a rise in the high-frequency signals. Additionally, the distribution range of signals in the parameter correlation plot expands progressively. With increasing shear stress, the AE count, amplitude, and energy also rise gradually. And the emergence of continuous high-frequency signals is noted. During the failure stage, numerous microcracks initiate and propagate within the specimen, with signal amplitudes ranging between 40 and 90 dB. The peak frequency range of the AE signals broadens, with high-frequency components mainly concentrated between 350 and 450 kHz. Loading tests conducted at shear displacement rates of 0.25–1.5 mm/min reveal a strong correlation between the AE count and the shear displacement rate. Furthermore, prior to the shear failure of the active waveguide structures, the AE count shows a positive correlation with shear displacement. After the shear failure of the waveguide structure specimens, the AE count gradually decreases from a higher level to a lower level, demonstrating a negative correlation with shear displacement. The active waveguide structure can monitor the internal deformation conditions of the bedding rock slope so as to provide some reference for the early warning research. In addition, quantitative statistical analysis and curve fitting are conducted on the relationship between AE statistical count and shear displacement under different loading rates. The measured data show good agreement with the fitted curves, and a distinct two-stage evolutionary pattern (positive correlation before peak and negative correlation after peak) is quantitatively identified. These results further enhance the reliability of using AE parameters for quantitative evaluation of shear failure characteristics and displacement rate effects in bedding rock slopes.

## 1. Introduction

Slope geological hazards pose significant threats to life and property security. As one of the most prevalent geological disasters, slope instability remains a critical area of research due to its potential for catastrophic losses. In recent years, acoustic emission technology has been increasingly utilized for slope monitoring, representing a significant advancement in the monitoring and early warning of geological hazards [1,2,3]. Subsequently, researchers have developed an active waveguide device that primarily generates AE signals through backfill materials [4,5,6]. This approach establishes a direct correlation between AE responses and slope deformation conditions. Compared to conventional AE monitoring methods, the active waveguide technique offers distinct advantages, including reduced signal attenuation, broader frequency coverage, and enhanced detection sensitivity. Consequently, it has garnered growing interest in engineering monitoring practices. Moreover, the AE signals can serve as a reliable reference for the early warning of slope geological hazards.

During the deformation and failure of rock slopes, acoustic waves propagate through the geotechnical medium. These acoustic signals provide valuable information for monitoring landslides and issuing early warnings [7]. However, Acoustic Emission (AE) signals are prone to attenuation and are influenced by environmental factors, which limit the accurate analysis of slope deformation conditions. To address this limitation, researchers have proposed the application of waveguide technology for monitoring slope stability. By capturing the evolutionary patterns of AE signals, this method can reflect the deformation characteristics of the slopes. Consequently, waveguide technology has garnered increasing attention. This technology facilitates effective monitoring of the deformation behavior of slope media, enabling precise identification of the deformation state and stress evolution characteristics of the internal mass of the slope [8,9,10]. Some researchers have utilized waveguide technology to investigate slope stability and to explore the relationship between AE parameters and slope displacement characteristics [11]. From a technical application perspective, researchers have suggested embedding a waveguide structure to achieve continuous monitoring of slope deformation [12]. In the 1990s, British scholar Smith first adopted steel bars as waveguide rods to monitor the deformation evolution of slopes [13,14]. Additionally, other researchers have embedded waveguide rods into soil slopes and backfilled the surrounding sand to enable real-time monitoring of slope stability [15,16]. With the increasing adoption of waveguide rods, researchers have further optimized waveguide monitoring devices by implementing active waveguide systems. These systems integrate multi-source data for comprehensive monitoring of slope stability and have been utilized in field experimental studies [17]. Deng et al. examined the variation characteristics of active waveguide measurements through model experiments, aiming to uncover the progressive evolution mechanisms of landslides. Moreover, combined with machine learning algorithms, these improvements can facilitate the automated classification of landslide movement patterns [18,19,20,21]. Based on an enhanced AE monitoring system, Cheon Dae-Sung, Byun Yo-Seph et al. [22,23] systematically investigated the evolutionary characteristics of AE parameters during slope deformation. The findings provide valuable insights for the engineering application of active waveguide technology. Additionally, Zhang et al. elucidated the evolutionary patterns of the AE signals from the active waveguide model, identified relevant early warning indicators, and established corresponding early warning criteria [24]. Xie et al. designed a physical model experiment to simulate the deformation of typical shear planes within slopes. Through a comprehensive analysis of displacement data, AE monitoring data, and time-frequency characteristics, they explored the evolutionary patterns of AE signals during shear deformation [25]. Chen et al. conducted model experiments on active waveguide structures to investigate the correlations among shear force, cumulative AE counts, and deformation. This research serves as a significant reference for the practical application of active waveguide technology [26]. Rock slope failure is not an abrupt event; it is a progressive process induced by the accumulation of displacement and deformation over time. In the initial stages of slope destabilization, microscale displacement and deformation occur within the rock mass, which subsequently triggers macroscopic instability and eventual failure [27,28]. The active waveguide monitoring technique can effectively capture microscale deformations within rock masses. By monitoring AE responses during the displacement and deformation processes, the method can elucidate the evolutionary mechanisms of slope instability, making it an essential tool for monitoring and providing early warnings of rock slope hazards [9,12,22,29,30]. However, most existing studies on active waveguide monitoring focus on steel rods embedded in loose granular backfill for soil slope monitoring, while relevant investigations targeting mortar-encapsulated steel waveguide structures adapted to bedding rock slopes are still insufficient. Previous literature mainly explored the AE response of granular-filled waveguide under overall sliding deformation, yet few works systematically reveal the stage-by-stage acoustic emission evolution law of mortar-bonded waveguide under shear dislocation of rock strata, lacking quantitative correlation analysis between AE parameters and shear displacement/displacement rate. Therefore, it is imperative to further investigate the AE characteristics during shear deformation of mortar-encapsulated waveguide to establish a solid theoretical and practical foundation for the early warning of bedding rock slope shear failure.

This study focuses on the monitoring principle of active waveguide structures in the bedding rock slope. The experiments were conducted under shear loading until the active waveguide structure failure. This research systematically investigates the AE characteristic parameters, parameter correlations, time–frequency features, and evolution patterns of AE signals during the failure process of the active waveguide structure. Furthermore, the correlation between AE count parameters and displacement rate is further explored, which can provide a fundamental reference for the monitoring and early warning research of rock slope hazards.

## 2. Monitoring Principle of Active Waveguide Structure

**Figure 1:**
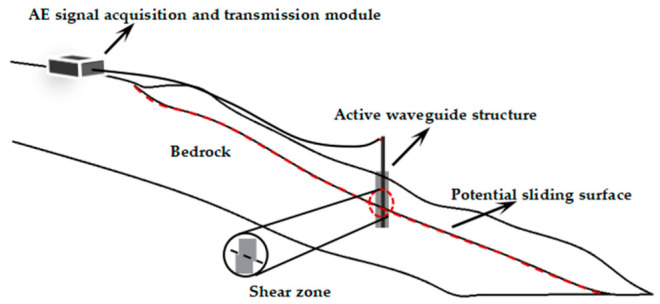
Schematic diagram of the monitoring method for the bedding rock landslide.

The evolutionary process of bedding landslides is influenced by the characteristics of the rock mass. The formation of weak interlayers creates the sliding plane within the rock mass. Over extended geological periods, the shear strength at the interlayer interfaces gradually decreases, leading to a continuous reduction in interlayer friction resistance. Consequently, the bedding rock masses tend to slide downwards. Once the sliding force exceeds the resisting force provided by the weak interlayers, the slope may experience slow creep deformation, which can ultimately result in slope failure. During the monitoring procedure, boreholes should be strategically arranged along the slope mass, with drilling extending to the stable bedrock. Waveguide rods are installed within these boreholes, followed by the injection of coupling mortar into the surrounding space. AE sensors are then placed at the top of the waveguide rods to capture signals generated by the deformation and fracture of the mortar inside the boreholes. It should be clarified that the active waveguide investigated in this study uses cement mortar as the acoustic emission source material, rather than the granular backfill. The evolution of these signals effectively reflects the deformation and displacement conditions of the rock strata. A detailed monitoring implementation scheme is presented in Figure 1.

**Figure 2:**
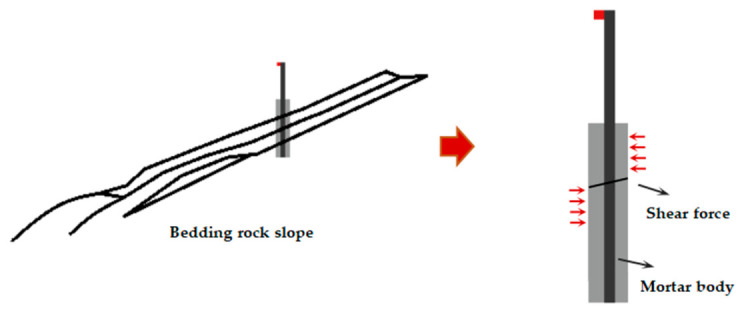
Mechanical characteristics of the waveguide structure with mortar materials.

During the sliding of bedding rock slopes, significant shear displacement occurs between adjacent rock strata. The AE signals are primarily generated by the mortar medium fracturing within the waveguide structure, resulting from the shear actions of the surrounding rock layers. As illustrated in Figure 2, the dislocation displacement of the rock strata exerts shear stress on the mortar body, leading to crack initiation and progressive coalescence, thereby generating intense AE signals. Shear failure tests on active waveguide structures can effectively simulate the loading response of these structures during slope deformation, establishing a direct correlation between the characteristics of AE signals and the slope deformation state.

## 3. Physical Experimental Design

**Figure 3:**
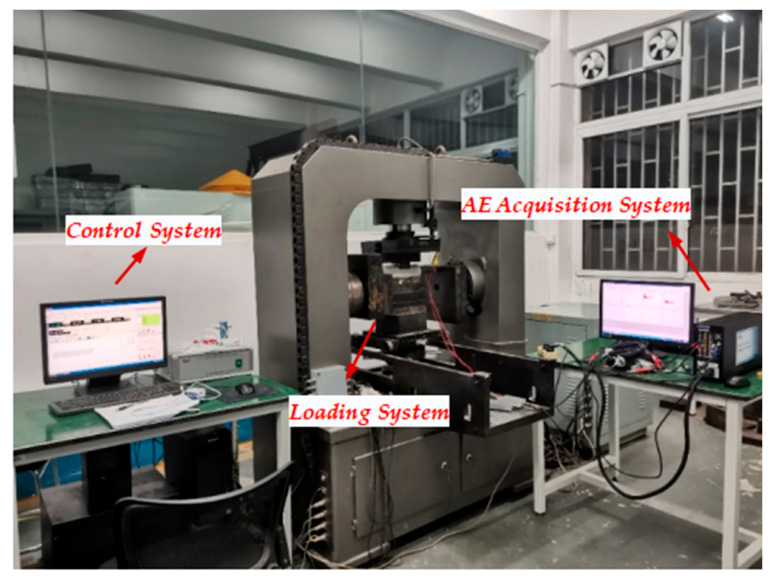
Test system.

Shear failure tests were conducted on active waveguide structures. The experiment mainly comprises a loading system with displacement measurement modules and the AE monitoring system, as shown in Figure 3.

### 3.1. Loading System

The experiment adopted the WDAJ-600 rheological testing machine, which is specially developed for axial compression and shear tests. This machine was manufactured by Changchun Kexin Test Instrument Co., Ltd., Changchun, China. Incorporated with advanced intelligent control technology, the apparatus allows both fast and slow loading under various working conditions. It is capable of measuring key mechanical parameters of specimens, including shear strength and compressive strength. The testing system is mainly composed of a control unit, multi-channel static strain measuring devices, and other auxiliary components.

### 3.2. AE Acquisition System

The system is a PCI-2 eight-channel device, designed to acquire AE parameters such as count, energy, amplitude, and frequency-domain information during the fracture process of active waveguide structures. This instrument was manufactured by Physical Acoustics Corporation, Princeton Junction, NJ, USA. Meanwhile, it can synchronously record the full waveforms of AE signals. The PCI-2 AE system is equipped with a noise reduction board, which effectively suppresses environmental interference and reduces noise contamination during signal acquisition. In this test, the AE sampling frequency is set to 1 MHz, with a threshold of 40 dB. The AE sensor used in the experiment is the Nano-30 to accurately capture AE signals. To improve signal transmission efficiency, petroleum jelly is applied as a coupling agent to the interface between the sensor and the waveguide rod.

It is worth noting that since all AE parameters (such as count, energy, amplitude, and duration) are derived from the same underlying physical waveforms captured by the piezoelectric sensor, any fluctuation in coupling conditions or sensor installation errors will inevitably and systematically affect all extracted parameters, including the AE count. However, in the context of multi-group parallel shear tests under varying loading rates, the statistical trend of the cumulative AE count exhibits high engineering repeatability. This is because the cumulative count reflects the macro-frequency of threshold-crossing pulses generated by the continuous fracture of the mortar medium, making it statistically robust for trend-fitting and displacement rate calibration, even when signal amplitudes suffer minor systematic attenuation due to coupling variations.

### 3.3. Active Waveguide Structures

**Figure 4:**
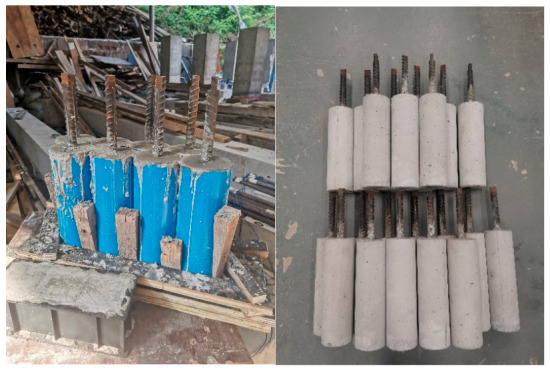
The prepared test specimens.

Active waveguide structures were fabricated using cement mortar for laboratory testing. The cylindrical mortar specimens measure 260 mm in length and 80 mm in diameter. A steel rod, with a diameter of 18 mm and a length of 400 mm, was longitudinally embedded at the core of each mortar specimen to function as the waveguide. The detailed configuration is depicted in Figure 4.

**Figure 5:**
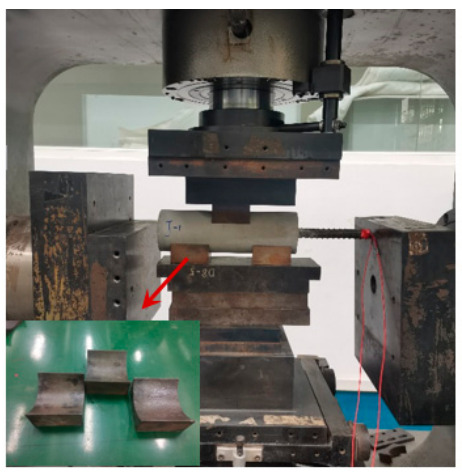
Shear failure test.

During the sliding deformation of bedding rock slopes, the active waveguide structures are mainly subjected to shear loading induced by the relative displacement of rock strata, which causes shear failure of the active waveguide structures. To trigger shear failure of the active waveguide structures, a rigid module made of Q345 steel plate was adopted for the test. This module was manufactured by Shougang Group Co., Ltd., Beijing, China. The experimental loading configuration is illustrated in Figure 5.

The test utilized displacement loading, with the loading rate ranging from 0.25 to 1.50 mm/min. The acoustic emission characteristics of the active waveguide structure were examined under different shear displacement rates. Three groups of parallel experiments listed in Table 1 were conducted, and the most representative group was selected for analysis.

## 4. Results and Analysis

### 4.1. Analysis of Failure Characteristics of Active Waveguide Structures

**Figure 6:**
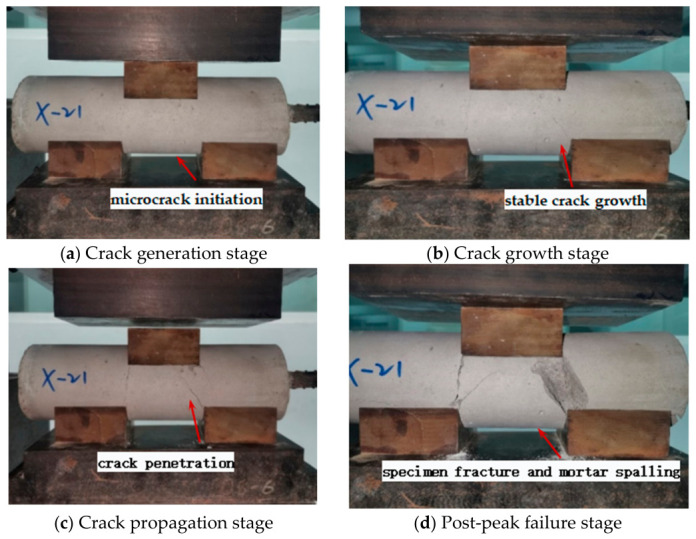
Failure patterns of the mortar material samples.

Based on shear failure tests of active waveguide structures, the test investigates the AE characteristics of specimens under shear loading and provides a reference for evaluating the state of bedding rock slopes. The shear failure process of active waveguide structures under shear loading is illustrated in Figure 6.

At the crack generation stage, microcracks gradually develop within the mortar without forming visible macroscopic fractures. Consequently, only a limited number of acoustic emission events are detected. During the stable crack growth stage, internal cracks in the mortar propagate steadily. AE signals occur more frequently in this stage, although no distinct macroscopic cracks are observed. In the accelerating crack propagation stage, a substantial number of microcracks emerge within the specimen, and the crack growth rate increases significantly. Cracks rapidly extend and merge throughout the specimen, accompanied by intense AE activity. In the post-peak failure stage, noticeable spalling of the mortar occurs, leading to shear failure of the specimen.

### 4.2. The AE Characteristics of the Shear Failure Process

During the sliding deformation of bedding rock slopes, the active waveguide structures are primarily subjected to shear loading induced by relative displacement between rock strata, which eventually leads to complete shear failure. Through shear failure tests on active waveguide structures, the evolutionary characteristics of AE signal parameters are systematically investigated.

#### 4.2.1. Analysis of AE Characteristic Parameters

**Figure 7:**
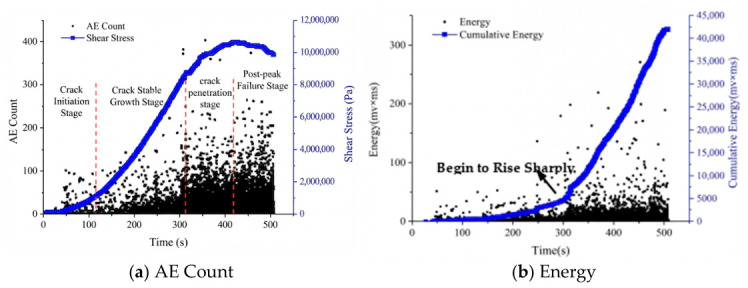

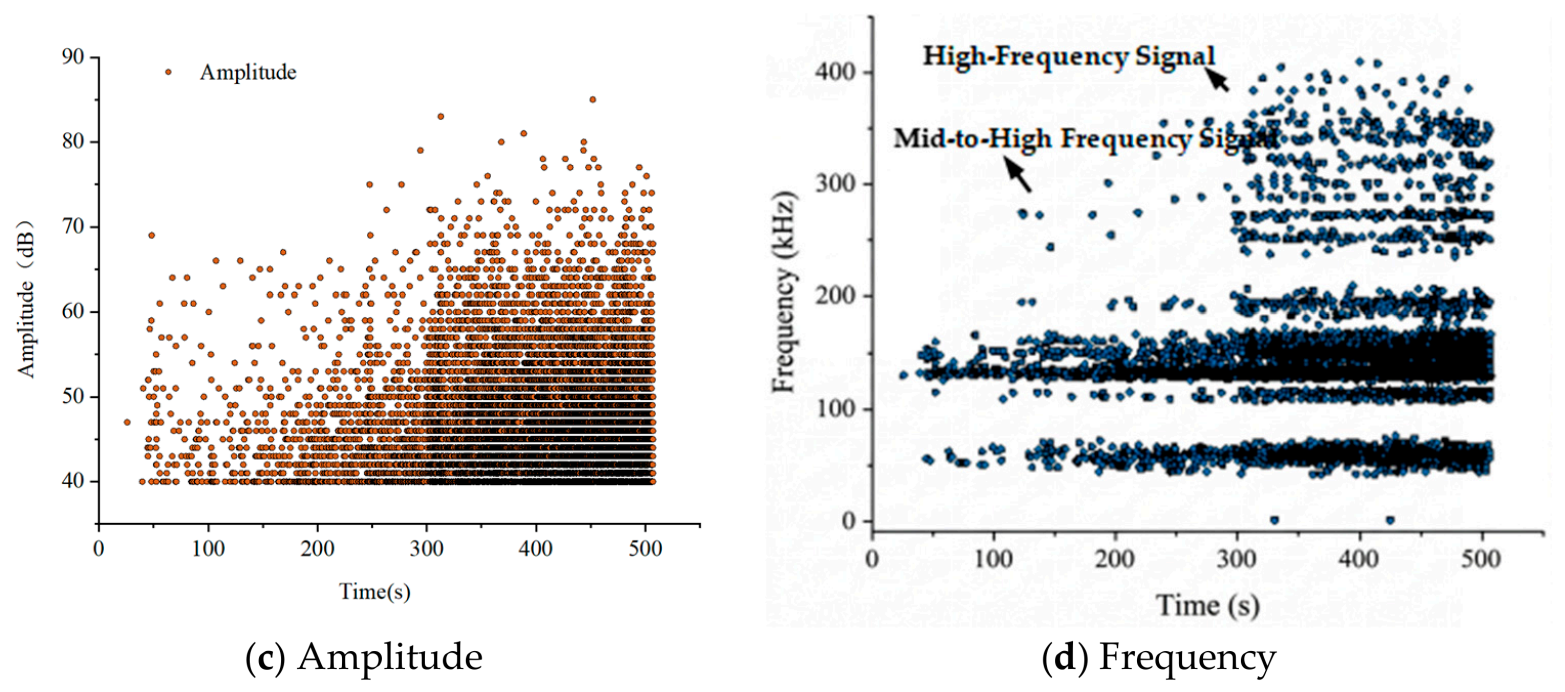
AE parameter characteristics.

As illustrated in Figure 7, the shear failure process of active waveguide structures generally consists of four stages: crack initiation stage (Stage I), crack stable growth stage (Stage II), crack penetration stage (Stage III), and post-peak failure stage (Stage IV). Crack initiation stage (Stage I): Shear stress is less than 15% of peak strength. AE activity remains extremely low with scattered discrete signals, and no visible microcracks appear on the specimen surface; only sporadic tiny fractures form inside the mortar. Crack stable growth stage (Stage II): Shear stress ranges from 15% to 90% of peak strength. The AE count rises steadily with continuous low-frequency signals, and internal cracks propagate uniformly without obvious visible fractures on the exterior. Crack penetration stage (Stage III): Shear stress exceeds 90% of peak strength until the peak value. The AE count surges sharply, accompanied by massive high-frequency signals concentrated at 350–450 kHz; internal microcracks rapidly coalesce, and tiny macroscopic cracks can be observed on the specimen surface. Post-peak failure stage (Stage IV): Shear stress gradually declines after reaching the peak. The AE count decreases from the maximum value, obvious mortar spalling occurs, and a fully penetrated main shear crack forms, leading to the complete shear failure of the specimen. Upon the application of shear loading, microcracks initiate and develop within the mortar without the emergence of visible macroscopic fractures. During this stage, acoustic emission activity remains relatively low, accompanied by a minimal count of AE events. As the shear load increases, internal cracks begin to propagate steadily. Correspondingly, AE activity intensifies, and the AE count rises consistently. When the shear stress exceeds 90% of the peak strength, a significant number of microcracks are generated within the specimen. These microcracks propagate rapidly and coalesce, ultimately leading to the macroscopic failure of the mortar. In the crack penetration stage, severe internal damage accumulates within the mortar, accompanied by vigorous AE activity and a sharp increase in AE count. Subsequently, shear stress gradually declines, leading to noticeable spalling in the mortar, and the specimen ultimately exhibits a fully fractured failure mode.

The peak frequency of AE signals is predominantly distributed in the low-frequency band during the early loading stage, with the frequency domain mainly concentrated below 200 kHz. At this stage, the proportion of peak frequency signals is approximately 23%. As shear stress increases, the activity of microcracks within the mortar propagates more extensively. At this stage, some AE signals emerge within the frequency range of 200–350 kHz. At this stage, the proportion of peak frequency signals is approximately 51%. As the specimen nears failure, numerous microfractures appear within the mortar and propagate rapidly. The energy accumulated during the early loading stage is released, resulting in a broad frequency distribution, with high-frequency signals primarily concentrated in the range of 350–450 kHz. At this stage, the proportion of peak frequency signals is approximately 26%.

Statistical analysis of high-frequency AE signals ranging from 300 kHz to 350 kHz shows that they account for 45% of all high-frequency AE events.

Regarding the amplitude and energy of AE signals, the signal amplitude is mainly distributed between 40 and 70 dB during the initial loading stage, while the cumulative energy curve rises gradually. As shear stress progressively increases, both AE amplitude and activity demonstrate a continuous upward trend. The signal amplitude ranges from 40 to 80 dB, with the majority concentrated in the 40–70 dB range. When shear stress approaches its peak, microcracks within the mortar enter an unstable propagation phase. Concurrently, the cumulative energy curve rises sharply, and the signal amplitude predominantly spans from 40 to 85 dB. Following this, cracks continue to propagate and coalesce, ultimately resulting in shear failure of the mortar and the formation of a distinct macroscopic fracture.

#### 4.2.2. The AE Characteristics Under the Influence of Shear Displacement Rate

**Figure 8:**
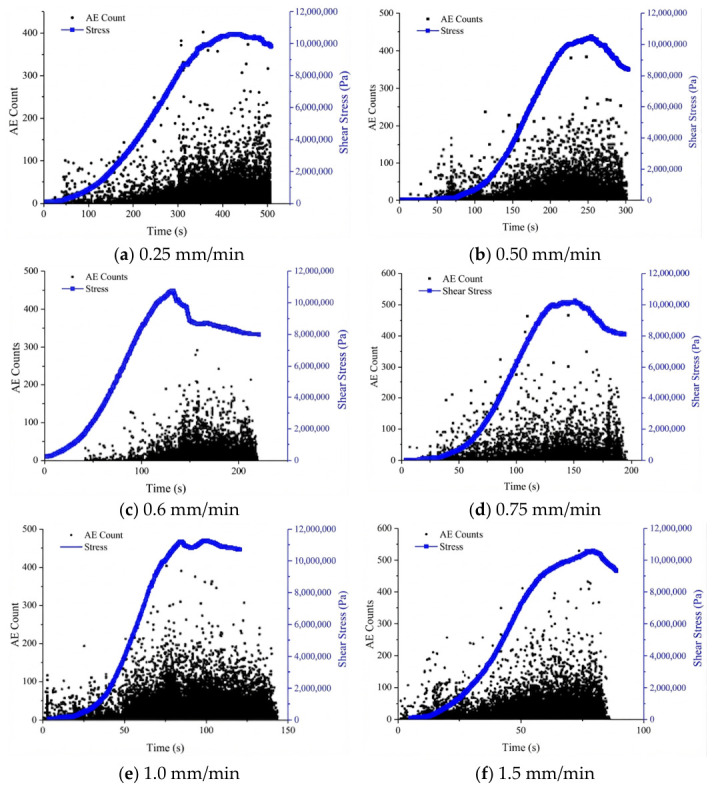
Evolution characteristics of AE count.

As illustrated in Figure 8, microcracks within the mortar develop rapidly with an increase in shear displacement rate. The microcracks in the mortar expand swiftly under shear stress. The specimen reaches its peak stress in a brief period, followed by the rapid formation of macroscopic shear cracks and subsequent shear failure. The acoustic emission count peaks at maximum shear stress and then decreases significantly, indicating that the generation and propagation of microcracks are diminished during the post-peak stage.

#### 4.2.3. Correlation Analysis of AE Parameters

**Figure 9:**
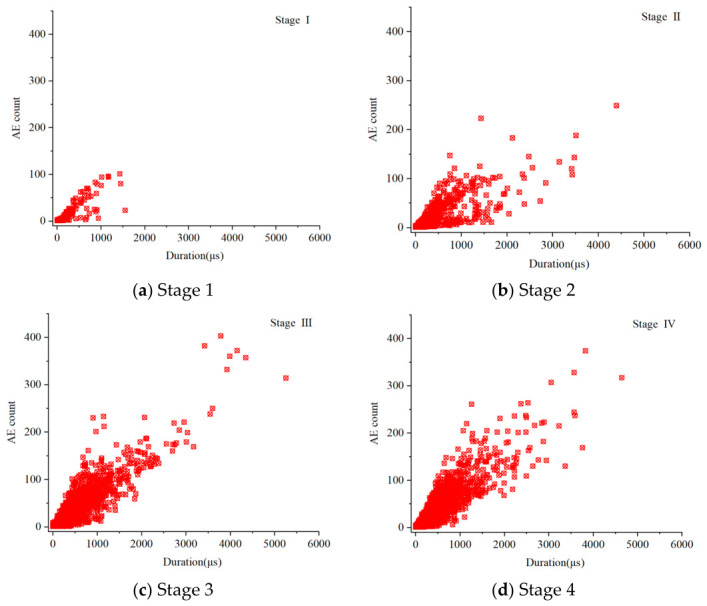
Evolution process of AE duration-count.

Figure 9 illustrates the relationship between the duration and count of acoustic emission signals. During the initial phase of shear loading, AE activity is relatively low, with both the duration and count of AE signals remaining at minimal levels, resulting in all data points in the correlation plot being concentrated within a narrow range. As crack propagation occurs, both the duration and count of AE signals increase continuously, leading to a gradual expansion of the distribution range of data points. As the specimen approaches its peak failure state, there is a significant rise in the duration of AE signals, accompanied by a continual increase in AE count. Consequently, the data points become widely dispersed and exhibit a random distribution over a broad range.

**Figure 10:**
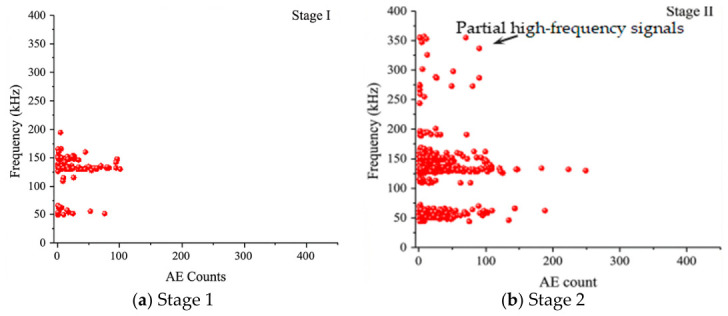

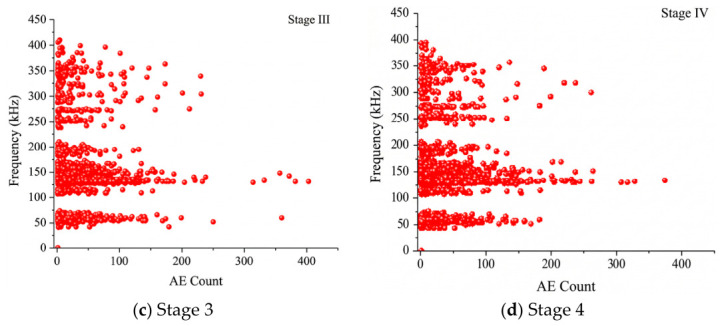
Evolution process of AE count-peak frequency.

Figure 10 illustrates the correlation between acoustic emission count and frequency domain distribution under shear loading. During the initial loading stage, AE activity remains relatively low. The peak frequency spectrum exhibits two distinct low-frequency bands, with the majority of frequency signals concentrated around 50 kHz and 150 kHz. As shear stress increases, the AE count rises, and the peak frequency range expands. A limited number of medium to high-frequency signals appear, primarily distributed within the range of 250 kHz to 350 kHz. As the specimen approaches the peak failure stage, the AE count significantly increases, accompanied by a prevalence of high-frequency signals predominantly concentrated between 350 kHz and 450 kHz.

**Figure 11:**
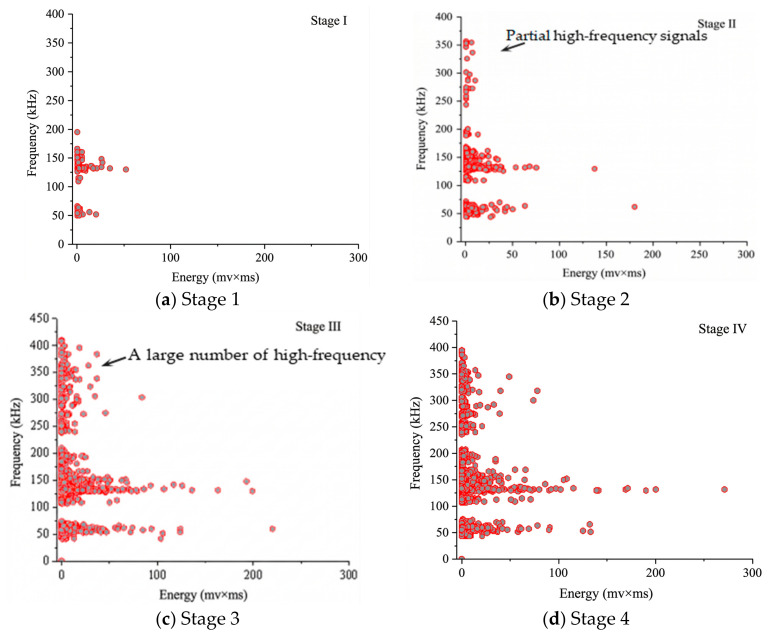
Evolution process of AE energy-peak frequency.

Figure 11 illustrates the correlation between acoustic emission signal energy and the frequency domain. During the initial loading stage, the AE signal energy is relatively low, with the frequency domain primarily concentrated within the range of 0–200 kHz. The signals predominantly cluster around the 50 kHz and 150 kHz bands. In the stage of stable crack growth, a limited number of high-frequency signals emerge, specifically within the range of 250–350 kHz. As the specimen approaches the peak failure stage, the AE signal energy increases significantly. The AE signals exhibit a marked rise, and the overall frequency domain range expands further. A substantial quantity of high-frequency AE signals is generated, with the high-frequency components primarily concentrated between 350 kHz and 450 kHz.

#### 4.2.4. Correlation Analysis

Based on a series of shear tests, the AE count parameters were statistically analyzed at a time interval of 10 s. The evolutionary trends of AE count statistical parameters are systematically investigated.

**Figure 12:**
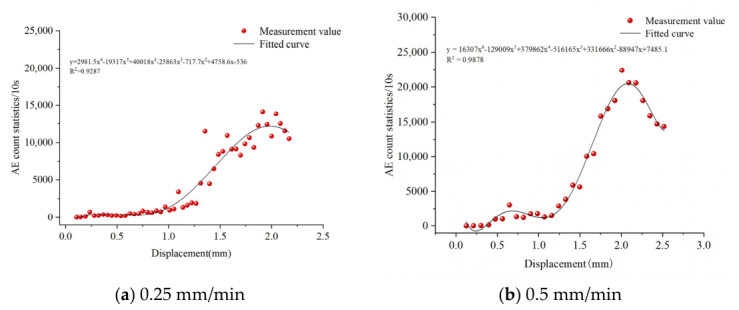

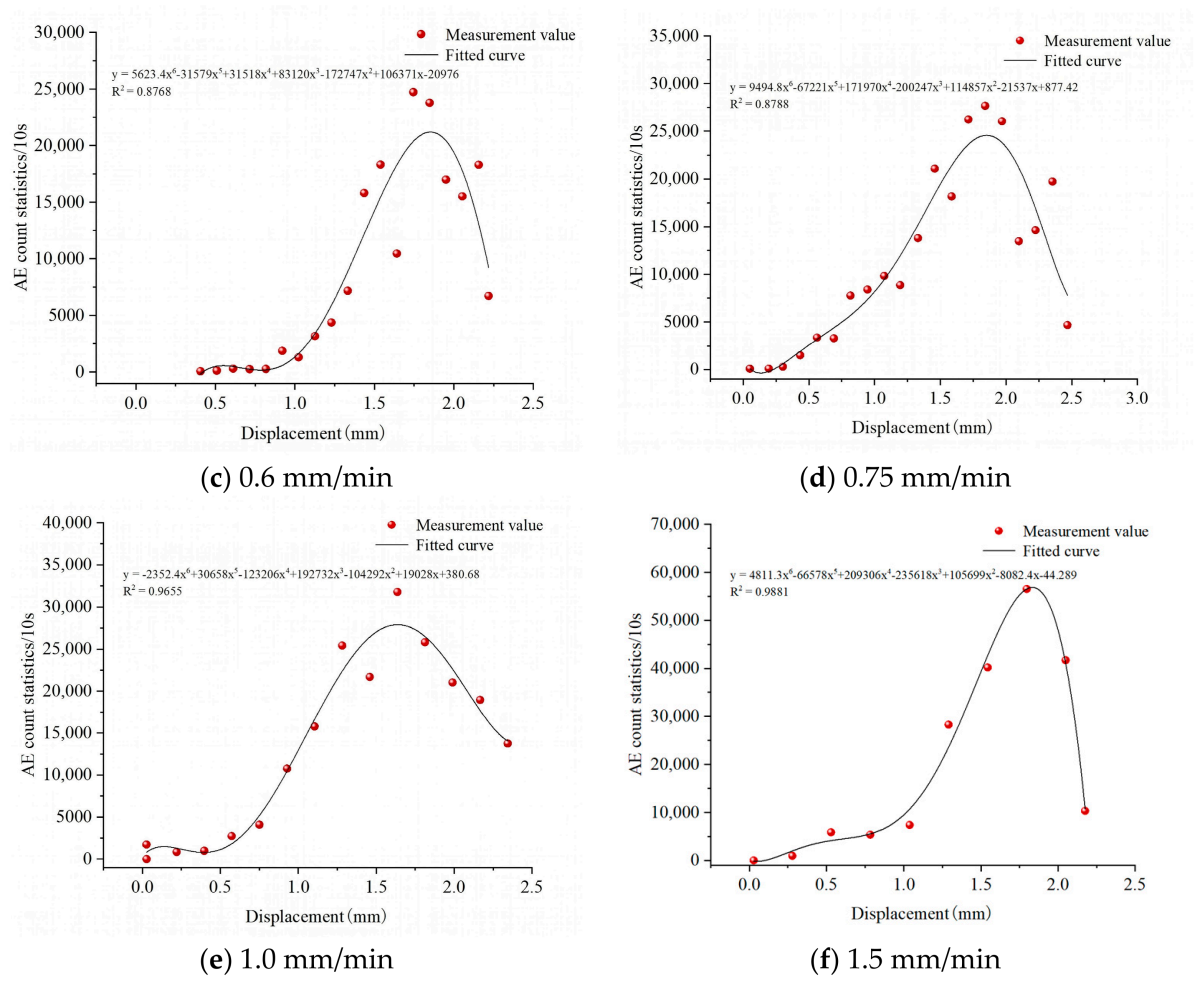
Correlation analysis between shear displacement and AE statistical count.

Figure 12 illustrates a strong correlation between shear displacement and AE count statistical parameters. During the initial stage of shear loading, microcracks initiate within the shear zone, producing a limited number of AE signals. As shear displacement gradually increases, the shear stress acting on the specimen correspondingly rises. The microcracks within the mortar propagate and coalesce rapidly, leading to an increase in AE activity. Consequently, the AE count statistical parameters exhibit a positive correlation with shear displacement. When the shear displacement reaches approximately 1.8 to 2.0 mm, a significant number of macroscopic cracks within the mortar. The rapid propagation and coalescence of microcracks ultimately result in the shear failure of the specimen. As the specimen nears the peak failure stage, the activity of internal fracture events diminishes noticeably, and the AE count statistical parameters decline significantly, indicating a negative correlation with shear displacement. This observation demonstrates that the specimen has transitioned into a fully shear failure state. Figure 12 presents the correlation analysis between shear displacement and AE statistical counts under six different displacement rates ranging from 0.25 to 1.5 mm/min (subplots a to f). In each plot, the red scatter points denote the actual measurement values, while the solid lines represent the corresponding fitted curves. The overall evolutionary trend distinctly shows a two-stage process: initially, the AE count exhibits a positive correlation with shear displacement, increasing continuously as internal microcracks propagate rapidly. When the shear displacement reaches approximately 1.8 to 2.0 mm, approaching the peak failure stage of the specimen, the AE count begins to decline significantly, demonstrating a negative correlation. The measured data under different loading rates are in good agreement with the fitting curves, which indicates that the fitting method can better describe the evolution characteristics of AE statistical count under different loading rates and provides a reliable basis for quantitative analysis of the influence of loading rate on failure characteristics.

**Figure 13:**
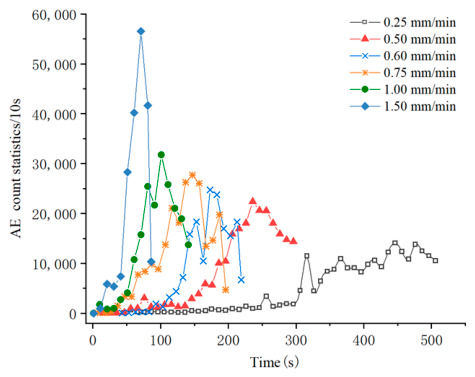
The evolution characteristics of AE statistical count under series displacement rates.

Figure 13 illustrates the evolution of AE count statistical parameters at displacement rates ranging from 0.25 to 1.50 mm/min. The AE count statistical parameters gradually increase with the shear displacement rate. As the shear displacement rate rises, the shear stress within the waveguide mortar specimen escalates rapidly. This rapid increase in stress accelerates the internal cracking of the mortar, leading to a corresponding rise in the AE count statistical parameters. When the specimen nears its peak stress state, numerous microcracks form rapidly, causing the AE count statistical parameters to reach their maximum value at this stage. Subsequently, the activity of internal microfracture events diminishes, resulting in a reduction in the AE count statistical parameters. This phenomenon indicates that the specimen has experienced comprehensive shear failure.

**Figure 14:**
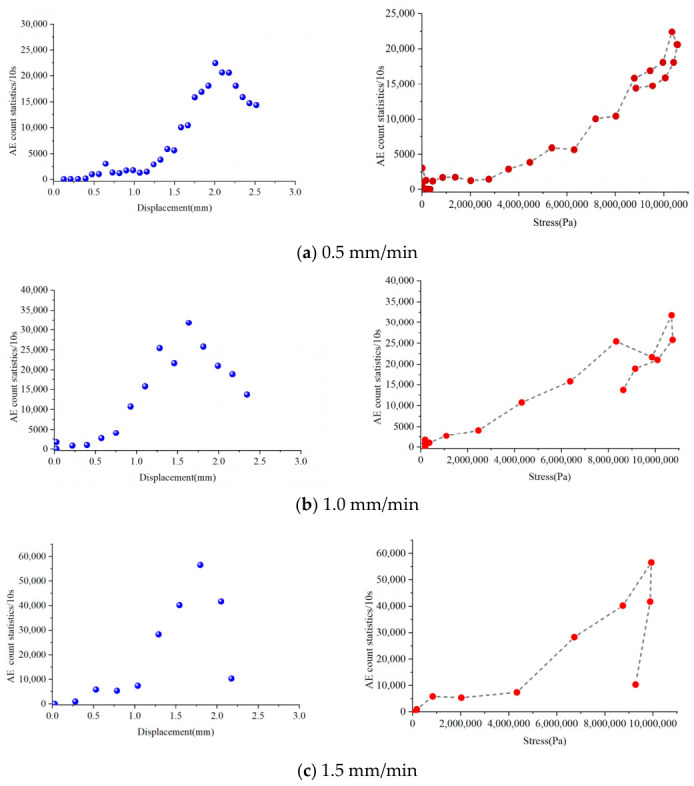
Evolution characteristics of AE statistics count vs. shear displacement or shear stress.

Figure 14 illustrates the relationships among the AE count statistical parameters, shear displacement, and shear stress at displacement rates of 0.5 mm/min, 1.0 mm/min, and 1.5 mm/min. As shear displacement and shear stress increase, the AE count statistical parameters gradually rise, demonstrating a positive correlation with both variables. With the continued development of shear deformation, shear stress reaches its maximum, accompanied by the rapid formation of internal microfractures within the specimen. Many of these cracks interconnect, ultimately resulting in macroscopic shear failure. At this point, the AE count statistical parameters reach their peak values. Following the shear failure of the specimen, shear stress decreases sharply. The AE count statistical parameters transition from a high level to a gradual decline, and the curve of AE count versus shear displacement exhibits a downward trend. Notably, the curve of AE count versus peak stress displays a distinct “reverse hook” shape.

#### 4.2.5. Time-Frequency Evolution Characteristics of AE Signals

**Figure 15:**
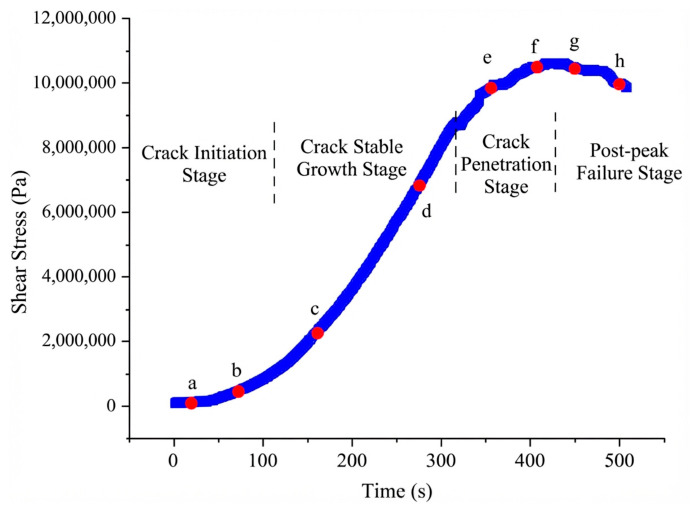
Characteristic points of AE signal time-frequency analysis.

Time–frequency analysis was conducted on AE signals acquired at different shear failure stages, which reveals the time–frequency evolutionary patterns throughout the entire specimen’s fracture process (Figure 15).

**Figure 16:**
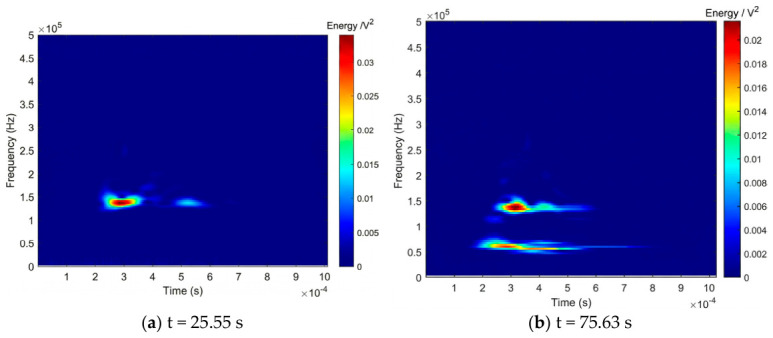

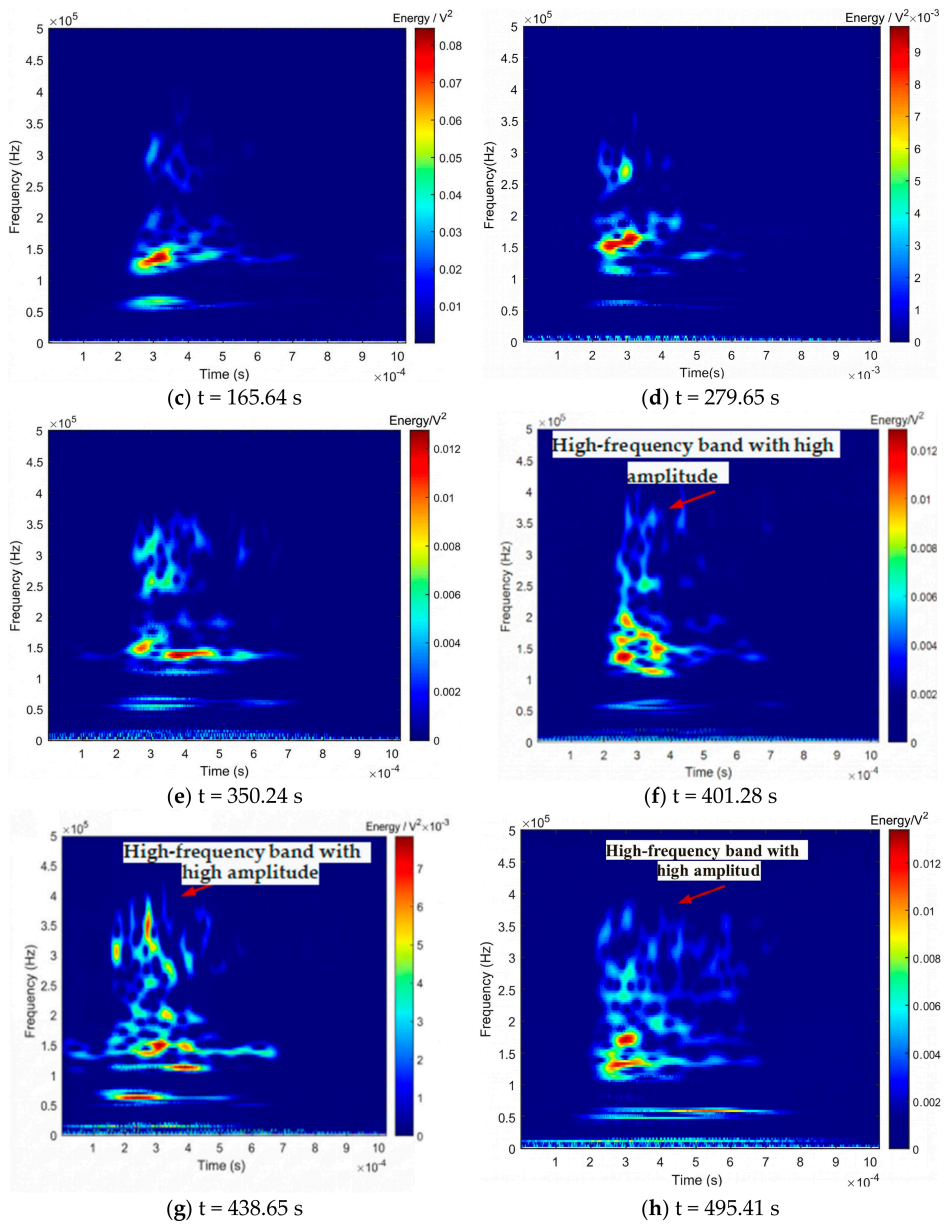
Evolution trend of AE signal time-frequency.

Figure 16a,b illustrate the time-frequency characteristics associated with the crack initiation stage. During this phase, the shear stress is relatively low, resulting in the formation of only a limited number of microcracks within the mortar. The time-frequency distribution exhibits a low-frequency characteristic, with signal energy predominantly concentrated in the 50–150 kHz band. Figure 16c,d present the time-frequency analysis obtained during the stable growth stage of the cracks. As the shear stress gradually increases, the internal microcracks propagate steadily under continuous loading. Although there is a slight increase in the energy within the high-frequency domain, its amplitude remains relatively low, and the dominant signal energy continues to be concentrated in the 50–150 kHz frequency range. Figure 16e,f correspond to the crack penetration stage. In this phase, the overall frequency range broadens significantly, with high-frequency components primarily distributed between 350 and 450 kHz. The distribution of frequency band energy indicates a relatively high amplitude. As the shear stress approaches its peak value, substantial microcracks quickly initiate and propagate within the mortar, ultimately leading to the formation of macroscopic shear cracks. The peak frequency range expands considerably, with numerous signals exhibiting high-frequency and high-amplitude characteristics, which suggest that the mortar specimen is nearing shear failure. Figure 16g,h illustrate the time-frequency characteristics associated with the post-peak failure stage, during which the amplitude of the high-frequency band energy remains at a relatively elevated level.

AE signal parameters can serve as reliable indicators for characterizing the evolution of shear failure in active waveguide structures. These parameters include AE count, cumulative energy curves, parametric correlations, and time-frequency characteristics of the signals. Additionally, the correlation curves of AE count statistical parameters with displacement and shear stress are considered. The evolutionary characteristics of these AE parameters under various shear failure modes in the specimens are summarized in Table 2.

This study selects AE count to characterize shear failure and establish fitting relationships with shear displacement. Existing studies prove that AE energy, amplitude, RA, AF and entropy can comprehensively evaluate material damage and identify tensile and shear cracking modes [31,32,33]. However, these parameters have obvious limitations in active waveguide monitoring of bedding rock slopes. As the adopted parameter, AE count has stable correlation with mortar microfracturing and adapts to low-power field monitoring with low computational cost. AE energy and amplitude are susceptible to sudden signal bursts, which reduce the accuracy of displacement fitting predictions [32,33]. Waveform attenuation and dispersion during waveguide propagation distort RA and AF values, failing to reflect real fracture characteristics for crack mechanism identification [31]. Although entropy can capture failure precursors with good noise resistance and quantify damage complexity, its complex calculation is unsuitable for long-term slope-monitoring hardware [32,33]. Overall, multi-parameter methods are reliable for laboratory material characterization. For field rock slope monitoring with active waveguides, AE count achieves the best balance of stability, fitting performance and engineering feasibility.

Comprehensive analysis reveals that few acoustic emission events are detected during the initial shear loading phase of active waveguide structures, with dominant signal frequencies primarily concentrated in the low-frequency range. As shear stress gradually increases, the data points in parametric correlation diagrams continuously expand, accompanied by a broader frequency range of AE signals and a gradual increase in the proportion of energy within the high-frequency domain. Furthermore, the shear stress increase triggers the rapid initiation and propagation of internal microcracks in the mortar, resulting in the generation of numerous AE signals. The peak frequency range is significantly expanded, and many signals exhibit high-frequency and high-amplitude characteristics, indicating that internal fracture activities are becoming more intense. Before the shear stress reaches its peak value, the statistical parameters of AE counts exhibit a positive correlation with the increase in shear displacement and shear stress. Following the occurrence of shear failure, AE count parameters consistently decline. Additionally, AE count statistics show a strong correlation with shear displacement rate, providing a theoretical foundation for the quantitative characterization of sliding displacement rates in bedding rock slopes. In practical monitoring of bedding rock slopes, a comprehensive analysis of the AE signal parameters can effectively assess the state of deformation evolution.

Although previous scholars have investigated the staged failure characteristics of geotechnical materials, the rising trend of AE count with load, and the correlation between AE parameters and deformation rate, the research carrier, test condition, analysis dimension and application object of this paper are essentially different from existing literature, and the core differentiated contributions are summarized as follows:

(1) Novel mortar-encapsulated waveguide specimen matching bedding rock slope engineering. Nearly all existing active waveguide studies adopt steel bars embedded in loose granular backfill, which are mainly designed for soil slope monitoring. Differently, this paper develops a fully mortar-bonded steel waveguide structure, taking solid cement mortar as the constraint medium instead of discrete granular filling materials. This structure matches the interlayer shear dislocation mechanical environment of layered rock mass and forms a unique crack generation and wave propagation mechanism. Few previous studies have carried out systematic full-process shear failure tests for this mortar-coupled waveguide form.

(2) Gradient multi-rate shear test design with complete parallel repeated specimens. Most related existing experiments only adopt a single fixed loading rate, lacking comparative analysis of AE response under variable shear velocities. This study sets six gradient shear displacement rates (0.25–1.50 mm/min), and three parallel repeated specimens are arranged for each loading rate as shown in Table 1. The test data can reflect the dispersion law of measured AE parameters and quantitatively reveal the coupling relationship between shear rate and full-stage AE evolution characteristics, which supplements the blank of multi-rate comparative tests in waveguide monitoring research.

(3) Multi-dimensional qualitative and quantitative joint analysis of full-process AE signals. Existing analogous studies mostly rely on simple qualitative descriptions of AE parameter variation trends and only divide failure stages by macroscopic phenomena. On this basis, this paper expands the analysis dimension: it systematically analyzes the staged distribution of AE count, amplitude, energy and peak frequency; constructs three types of parametric correlation scatter diagrams (duration-count, frequency-count, energy-frequency) for four failure stages; and conducts time-frequency spectrum extraction and feature analysis of characteristic fracture points. Meanwhile, the fitting relationship between AE count and shear displacement/stress under different shear rates is established, and the two-stage positive/negative correlation rule before and after peak shear stress is quantitatively summarized, rather than only relying on visual judgment of curve trends.

(4) Targeted high-frequency precursor warning indicators for rock interlayer shear slip. Existing waveguide early warning research mainly aims at sliding failure of soil slopes dominated by granular medium deformation. Targeting bedding rock slope interlayer shear failure, this paper extracts two exclusive precursor indicators based on test results: the concentration range of high-frequency AE signals (350–450 kHz) near peak stress and the reverse-hook characteristic of the AE count-shear stress curve. These two characteristic signals can serve as quantitative early warning criteria for layered rock mass shear instability and provide a targeted monitoring basis for rock slope engineering, which fills the gap of the AE early warning index system for mortar-encapsulated waveguides applied to rock slopes.

In summary, the novelty of this work lies in the special mortar-bonded waveguide specimen adapted to interlayer rock slope conditions, multi-gradient shear rate parallel test scheme, multi-dimensional time-frequency and parametric quantitative analysis system, and the extracted rock mass shear slip precursor warning indicators. These differentiated contents distinguish this study from the existing granular-filled active waveguide research focusing on soil slopes, single loading rate and simple qualitative analysis.

## 5. Conclusions

Based on the principle of active waveguide monitoring for bedding rock slopes, this paper analyzes the evolution characteristics of the acoustic emission signals under shear test conditions and explores the correlation between the AE statistical parameters and shear displacement. The main conclusions are as follows:

(1) Under the shear loading process, the AE count, amplitude, and energy increase with the shear stress progressively. Meanwhile, the peak frequency range of AE signals gradually expands, including some high-frequency signals within the range of 250–350 kHz. When the shear stress exceeds 90% of the peak stress, the active waveguide specimen enters the critical failure stage, which is accompanied by the initiation of extensive microcracks. In this stage, the AE signal amplitude ranges from 40 to 90 dB, and the high-frequency domain mainly concentrates within 350–450 kHz.

(2) At the initial loading stage, the AE signal points in the parameter correlation diagrams are concentrated within a relatively small range. As the specimen approaches the failure stage, the AE count rises sharply, and the energy amplitude of high-frequency bands increases significantly. Meanwhile, the distribution range of AE signal points expands gradually, which can be regarded as a typical precursor of the shear failure within active waveguide structures. Subsequently, the shear stress gradually decreases, and the active waveguide specimen generates a macroscopic shear fracture.

(3) A series of shear loading tests (conducted at 0.25–1.5 mm/min) demonstrates a strong correlation between AE count parameters and shear displacement rate, confirming a reliable quantitative relationship between the two variables. Prior to shear failure in active waveguide structures, AE count parameters exhibit a positive correlation with shear displacement. Following specimen failure, AE count parameters gradually decrease from a higher level, while the curve of AE count versus shear stress displays a distinct reverse-hook shape. These characteristics may provide useful insights for monitoring the internal deformation and failure of bedding rock slopes.

(4) Based on the quantitative statistical analysis of AE count parameters at 10 s intervals, fitting relationships between AE statistical count and shear displacement are established under six displacement rates (0.25–1.5 mm/min). The fitted curves are in good agreement with the measured data, clearly exhibiting a two-stage evolution law characterized by a positive correlation before peak stress and a negative correlation after peak stress. This quantitative characterization provides a reliable analytical framework for evaluating the influence of displacement rate on shear failure behavior and improves the applicability of AE-based early warning criteria for bedding rock slopes.

## Figures and Tables

**Table 1:** Testing groups of the displacement loading.

Loading Method	Loading Rate (mm/min)	Parallel Experiment Number		
Displacement Loading	0.25	B11	B12	B13
	0.50	B21	B22	B23
	0.60	B31	B32	B33
	0.75	B41	B42	B43
	1.00	B51	B52	B53
	1.50	B61	B62	B63

**Table 2:** Evolution characteristics of AE parameters.

AE Parameter Indicator Characteristics	Crack Initiation Stage	Crack Stable Growth Stage	Crack Penetration Stage	Post-Peak Failure Stage
AE Count	low level (8%)	increase gradually (25%)	increase rapidly (41%)	decrease gradually (26%)
Frequency Range	low-frequency	mid-to-high frequency	high-frequency domain (45%)	high-frequency domain (38%)
AE Duration-Count	a small range	to a large range	wide range	wide range
AE Amplitude-Energy	a small range	a larger range	wide range	wide range
AE -displacement Curve	rise	rise rapidly	rise steeply	drop back
AE—stress Curve	Rise	rise rapidly	rise steeply	reverse hook

## Data Availability

All data analyzed during this study are included in this published article.
